# P-Selectin preserves immune tolerance in mice and is reduced in human cutaneous lupus

**DOI:** 10.1038/srep41841

**Published:** 2017-02-02

**Authors:** Rafael González-Tajuelo, Javier Silván, Alicia Pérez-Frías, María de la Fuente-Fernández, Reyes Tejedor, Marina Espartero-Santos, Esther Vicente-Rabaneda, Ángeles Juarranz, Cecilia Muñoz-Calleja, Santos Castañeda, Carlos Gamallo, Ana Urzainqui

**Affiliations:** 1 Fundación de Investigación Biomédica (FIB), Instituto de Investigación Sanitaria-Princesa (IIS-Princesa), Hospital de la Princesa, Immunology Department, C/Diego de León 62, 28006, Madrid, Spain; 2 FIB, IIS-Princesa, Hospital de laPrincesa, Cytometry and Autoimmunity Unit, C/Diego de León 62, 28006, Madrid, Spain; 3 FIB, IIS-Princesa, Hospital de la Princesa, Rheumatology Department, C/Diego de León 62, 28006, Madrid, Spain; 4 Biology Department, Facultad de Ciencias, Universidad Autónoma de Madrid and Instituto Ramón y Cajal de Investigaciones Sanitarias (IRYCIS), C/Francisco Tomás y Valiente 7, 28049, Madrid, Spain; 5 Pathology Department, Facultad de Medicina, Universidad Autónoma de Madrid, C/Arzobispo Morcillo 4, 28029, Madrid, Spain

## Abstract

Mice deficient in P-Selectin presented altered immunity/tolerance balance. We have observed that the absence of P-Selectin promotes splenomegaly with reduced naïve T cell population, elevated activated/effector T cell subset, increased germinal center B and Tfh populations and high production of autoreactive antibodies. Moreover, 1.5-3-month-old P-selectin KO mice showed reduced IL-10-producing leukocytes in blood and a slightly reduced Treg population in the skin. With aging and, coinciding with disease severity, there is an increase in the IL17^+^ circulating and dermal T cell subpopulations and reduction of dermal Treg. As a consequence, P-Selectin deficient mice developed a progressive autoimmune syndrome showing skin alterations characteristic of lupus prone mice and elevated circulating autoantibodies, including anti-dsDNA. Similar to human SLE, disease pathogenesis was characterized by deposition of immune complexes in the dermoepidermal junction and renal glomeruli, and a complex pattern of autoantibodies. More important, skin biopsies of cutaneous lupus erythematosus patients did not show increased expression of P-Selectin, as described for other inflammatory diseases, and the number of vessels expressing P-Selectin was reduced.

Selectins (E-, L- and P-Selectin) mediate leukocyte rolling during their extravasation through interactions of their N-terminal lectin domains with a sialyl Lewis x (sLex) capping structure on leukocytic P-Selectin glycoprotein ligand-1 (PSGL-1)^1^,^2^. P-Selectin is stored in the α-granules of platelets and Weibel-Palade bodies of endothelial cells, and is rapidly mobilized to the membrane upon activation by complement, oxygen-derived free radicals or thrombin^3^,^4^,^5^,^6^, without requiring new protein synthesis. Additionally, TNF, IL-1β, or LPS increase also murine P-Selectin mRNA and protein in endothelial cells^7^,^8^,^9^,^10^.

Systemic lupus erythematosus (SLE) is a chronic, inflammatory autoimmune disease characterized by the production of autoantibodies against double strand DNA (dsDNA) and nuclear antigens, immune complex deposition, complement activation and polyclonal expansion of autorreactive lymphocytes^11^,^12^. SLE predominantly affects women (6–10:1 ratio of women to men) in the childbearing years^12^,^13^. Clinical manifestations of SLE include inflammation of the skin and internal organs, which are translated into non-specific symptoms like fever, arthralgia, skin rashes and anemia^12^. P-Selectin levels are elevated in the urine of SLE patients and correlate with disease severity^14^. Genome-wide linkage studies in humans have suggested an important role for P-Selectin in SLE. Indeed, the P-Selectin gene is located in the SLE linkage region on human chromosome 1 (1q23)^15^,^16^. Moreover, variations in the upstream region of P-Selectin are a risk factor for SLE, and two risk alleles have been identified potentially affecting the transcription of P-Selectin and the binding to P-Selectin glycoprotein ligand-1 (PSGL-1)^15^, the main ligand for P-Selectin expressed on all leukocyte subsets, and also a ligand for E- and L-Selectin^3^,^17^,^18^,^19^.

P-Selectin/PSGL-1 axis is involved in the generation of regulatory T (Treg) cells^20^. PSGL-1 null (*Psgl-1*^−/−^) mice have altered tolerance/immunity balance in the colonic lamina propria and skin, and spontaneously develop an autoimmune syndrome similar to human scleroderma^21^,^22^. Experimental disease models indicate that although mice lacking P-Selectin, E-Selectin or both are significantly protected from neutrophil-dependent injury^5^,^23^,^24^, selectin deficiency induced disease exacerbation in models of glomerulonephritis or collagen-induced arthritis, suggesting a protective role for endothelial P-Selectin in inflammation^5^,^25^,^26^. Numerous murine models of SLE have been described such as MRL/MpJ^faslpr^ (MRL/lpr), BSXB and NZB mice crossed with NZW strains (NZB/W)^27^, characterized by high levels of circulating autoantibodies, systemic vasculitis, lymphadenopathy, splenomegaly, skin and renal lesions and early death due to renal dysfunction, hypertension and spontaneous hemorrhage^27^,^28^. Studies performed with this SLE experimental models described that P-Selectin deficiency in lupus-prone mice resulted in more rapid development of glomerulonephritis and dermatitis and earlier death^29^ and that P-Selectin levels appear elevated in the urine of lupus-prone mice^14^.

Given all these data, we sought to determine whether *P-Sel*^−/−^ mice develop a connective tissue-related autoimmune syndrome sharing some characteristics with *Psgl-1*^−/−^ mice.

## Results

### Presence of circulating autoantibodies and augmented splenic reactivity in *P-Sel* ^−/−^ mice

**Figure 1:**
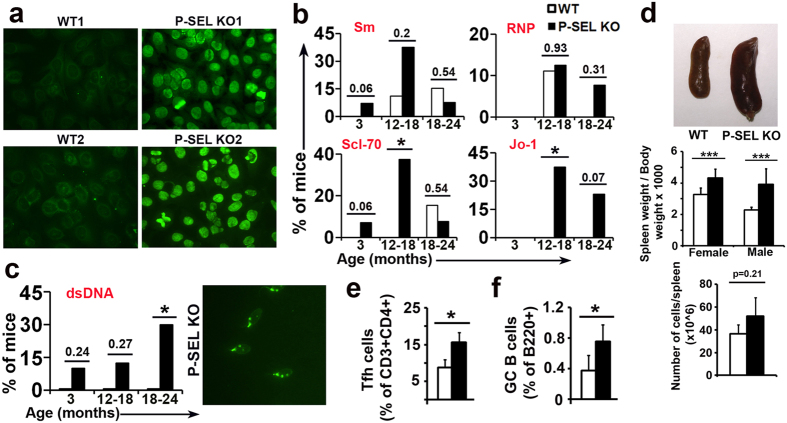
Spontaneous generation of autoantibodies related to connective tissue autoimmune diseases in *P-Sel*^−/−^ mice. (**a)** Representative immunofluorescence photomicrographs of HEp-2 cells incubated with serum from 2 independent wild-type (WT) and 2 independent *P-Sel*^−/−^ mice. (**b)** Percentage of mice positive for anti-Sm, RNP, Scl-70 and Jo-1 autoantibodies (n = 8–10 animals per group); *p < 0.05 by Chi-square test. (**c)** Percentage of mice positive for anti-dsDNA autoantibodies (n = 8–10 animals per group); *p < 0.05 by Chi-square test. Immunofluorescence photomicrographs of *C. luciliae* incubated with serum of a *P-Sel*^−/−^ mouse (right panel). (**d)** Photograph of representative spleens of 3-month-old WT and *P-Sel*^−/−^ mice (upper panel). Spleen weight/body weight ratio of female and male 3-month-old WT and *P-Sel*^−/−^ mice (middle panel) (n = 6 mice per group). Total number of cells per spleen of WT and *P-Sel*^−/−^ mice (lower panel). (**e**,**f)** Percentage of splenic follicular T helper (Tfh) cells (**e**) and germinal center (GC) B cells (**f**) in 3-month-old male WT and *P-Sel*^−/−^ mice. *p < 0.05; ***p < 0.005 by Student’s two-tailed t-test. Bars show the mean ± standard deviation (SD).

Sera from *P-Sel*^−/−^ mice contained anti-cytoplasmic and anti-nuclear autoantibodies with a speckled or mitotic pattern (Fig. 1a), but not anti-centromere autoantibodies. ELISA identified several antigens recognized by these autoantibodies, including topoisomerase I (Scl-70), U1-RNP, Sm and t-RNA synthetase (Jo-1) (Fig. 1b). Sera were also positive in the Crithidia assay for anti-dsDNA autoantibodies (Fig. 1c), a hallmark of human lupus erythematosus^1^,^3^,^5^, whereas none of the WT sera were positive in the same conditions. The anti-Sm, anti-topoisomerase I, and anti-dsDNA antibodies could be already detected at 3 months of age. The autoantibodies were co-expressed in the same animals and the percentage of mice with autoantibodies increased as a function of age, reaching a maximum at 12–18 months (Fig. 1b) and decreasing at 18–24 months, especially anti-Sm and anti-Scl-70 (Fig. 1b). The prevalence of anti-dsDNA autoantibodies increased gradually with ageing, from 10% in the 3-month-old group to 30% in the 18–24-month-old group (Fig. 1c). Consistent with this exacerbation of humoral immunity, we observed a remarkable spleen enlargement in both male and female 1.5-3-month-old *P-Sel*^−/−^ mice (Fig. 1d), which has been described previously in lupus-prone mouse strains^30^,^31^. Considering the increased cellularity of 3 month-old *P-Sel*^−/−^ spleens (Fig. 1d, lower panel), we analyzed the germinal centre (GC) B cell and the follicular helper T cell subsets (Tfh). We found an increase in both lymphocytic populations, indicating a more reactive state of *P-Sel*^−/−^ spleens (Fig. 1e,f).

### Immune homeostasis imbalance in *P-Sel* ^−/−^ mice blood and spleen

**Figure 2:**
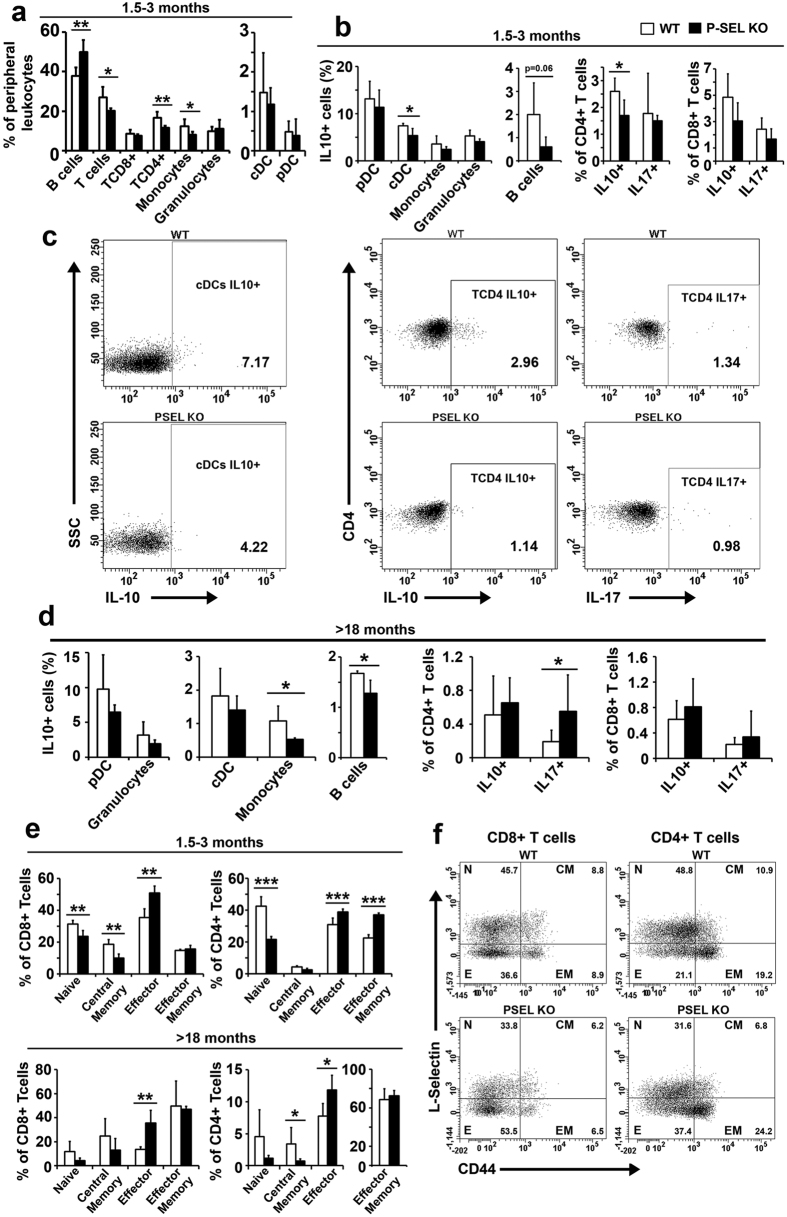
Peripheral blood and spleen immune system characterization in *P-Sel*^−/−^ mice. (**a)** Relative frequency of peripheral blood leukocyte populations of 1.5–3 month-old WT and *P-Sel*^−/−^ mice. (**b**,**d**) Percentage of IL-10^+^ conventional dendritic cells (cDC), plasmacytoid DC (pDC), monocytes, granulocytes and B cells; and frequency of IL-10 and IL-17 producing CD4^+^ and CD8^+^ T lymphocytes, in 1.5-month-old (**b**) and >18-month-old (**d**) WT and *P-Sel*^−/−^ mice. (**c)** Representative dot plots of IL-10^+^ cDCs and IL-10 and IL-17 producing CD4^+^ T cells in 1.5-months old WT and *P-Sel*^−/−^ mice. (**e)** Phenotyping of CD4^+^ and CD8^+^ splenic T lymphocytes according to the expression of the naïve/memory/effector markers CD62L and CD44 in 1.5–3 months-old (upper panels) and >18 month-old (lower panels) WT and *P-Sel*^−/−^ mice. (**f)** Representative dot plots showing the distribution of 1.5–3-month-old mice splenic populations according to the expression of L-Selectin and CD44. In all cases, n = 4 mice per group. In all cases, n = 4 mice per group. Bars represent the mean ± SD. *p < 0.05; **p < 0.01; ***p < 0.005, by Student’s two tailed t test.

The study of the peripheral blood leukocyte populations of 1.5-3-month-old male mice showed an important increase in the B cell compartment of *P-Sel*^−/−^ mice. In contrast, the T cell compartment was reduced, mainly due to the decrease of the CD4^+^ T cell subset. Additionally, the monocytic population was also reduced in *P-Sel*^−/−^ animals (Fig. 2a).

We studied the cytokine production of the circulating immune populations and we found a reduction in the percentage of IL-10^+^ producing cells of 1.5-3-month-old *P-Sel*^−/−^ mice, that was statistically significant among cDC and CD4^+^ T lymphocytes and close to the statistical signification in the B cell population (Fig. 2b,c, left panels). Circulating leukocytes of >18-month-old mice showed a statistically significant reduction in the percentage of IL-10^+^ producing monocytes and B cells (Fig. 2d, left and middle panels). Interestingly, the IL-17^+^ CD4^+^ (Th17) population was increased in aged *P-Sel*^−/−^ mice (Fig. 2d, right panels).

To evaluate the effector and memory state of T cells, we analyzed the expression of CD62L and CD44 splenic CD4^+^ and CD8^+^ T cells (Fig. 2e,f). Naïve (CD62L^+^CD44^neg^) and central memory (CD62L^+^CD44^+^) subsets were decreased in 1.5-month-old *P-Sel*^−/−^ CD8^+^ splenic T cells, while effector (CD62L^neg^CD44^neg^) CD8^+^ T cells were augmented. In the case of splenic CD4^+^ T cells, the naïve subset was severely diminished in the *P-Sel*^−/−^, while the effector (CD62L^neg^CD44^neg^) and effector memory (CD62L^neg^CD44^+^) compartments were highly increased (Fig. 2e, upper panels). Regarding >18-month-old mice, as compared with the WT mice, we found an increment in the rates of effector TCD8^+^ and TCD4^+^ lymphocytes, and a decrease of central memory TCD4^+^ lymphocytes in *P-Sel*^−/−^ mice (Fig. 2e, lower panels and 2f).

### Immune homeostasis imbalance in the skin of *P-Sel* ^−/−^ mice

**Figure 3:**
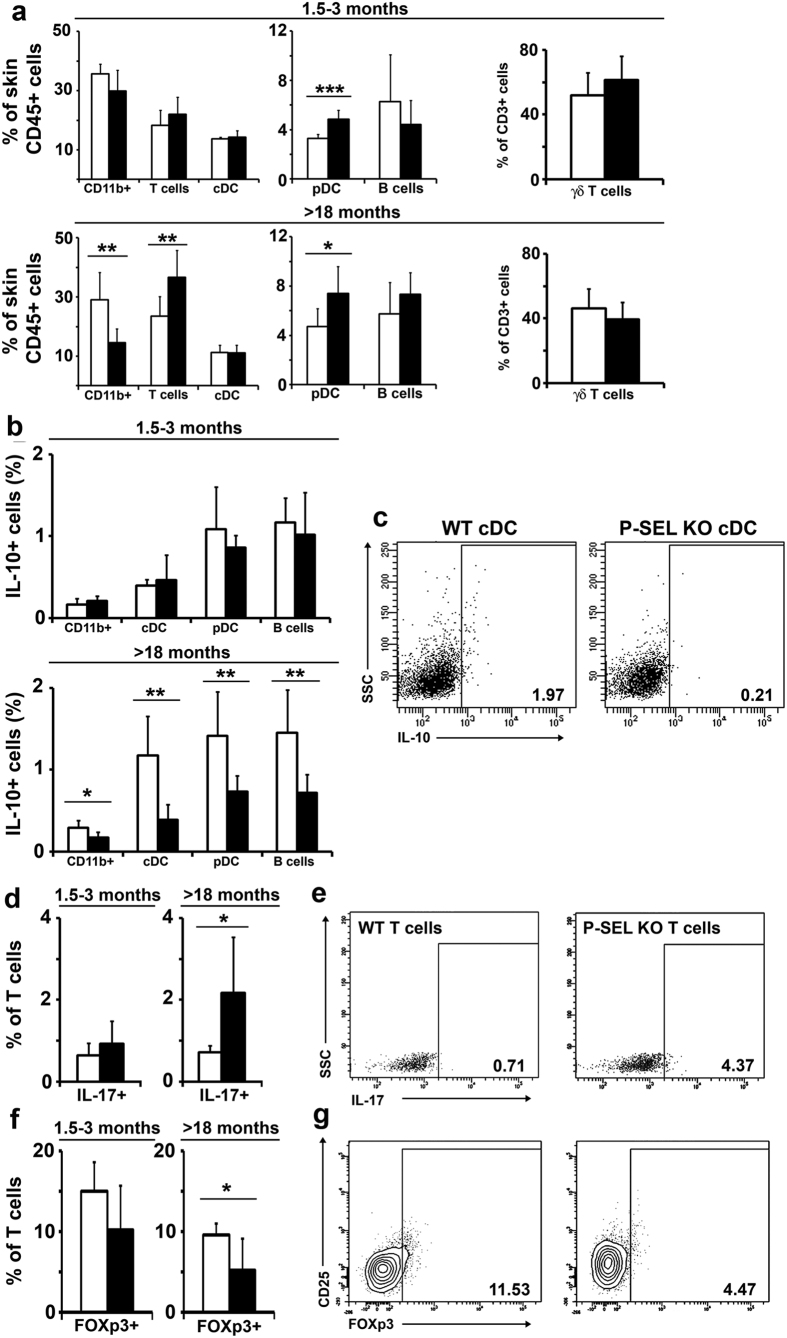
Characterization of the skin immune system of *P-Sel*^−/−^ mice. (**a)** Relative frequency of skin leukocyte populations of 1.5–3 month-old and >18-month-old WT and *P-Sel*^−/−^ mice. (**b)** Percentage of IL-10^+^ macrophages (CD11b^+^), conventional dendritic cells (cDC), plasmacytoid DC (pDC) and B cells, in 1.5–3-month-old and >18-month-old WT and *P-Sel*^−/−^ mice. (**c)** Representative dot plots of IL-10^+^ cDCs in >18-month-old WT and *P-Sel*^−/−^ mice. (**d)** Percentage of IL-17^+^ T cells in 1.5–3-month-old and >18-month-old WT and *P-Sel*^−/−^ mice. (**e)** Representative dot plots of IL-17^+^ T cells in >18-month-old WT and *P-Sel*^−/−^ mice. (**f**) Percentage of FOXP3^+^ T cells in 1.5–3-month-old and >18-month-old WT and *P-Sel*^−/−^ mice. (**g)** Representative dot plots of FOXP3^+^ T cells in >18-month-old WT and *P-Sel*^−/−^ mice. *p < 0.05; **p < 0.01 by Student’s two tailed t test. n = 6 mice per genotype.

Regarding the skin, one of the main organs affected in human lupus, we did not find significant changes in the different immune subpopulations in 1.5-3-month-old mice, except from an increased percentage of plasmacytoid DC (pDC) in *P-Sel*^−/−^ animals (Fig. 3a, upper panels). However, in aged animals, we found a reduced population of macrophages and a significant increment in the T cell and pDC subsets (Fig. 3a, lower panels). Among T cells, we did not find any difference in the percentage of gamma/delta (γδ) T cells (Fig. 3a, right panels). We studied the cytokine production of the different populations, and only the >18-month-old *P-Sel*^−/−^ mice showed a reduction in the percentages of IL-10^+^ macrophages, cDC, pDC and B cells (Fig. 3b, lower panels; Fig. 3c). Accordingly, aged knocked-out mice displayed a reduced FOXP3^+^ (Fig. 3d and e) and an increased Il-17^+^ T cell populations (Fig. 3f and g). We did not found significant differences in cytokine production in 1.5-3-month-old mice.

### Histological alterations in the skin of *P-Sel* ^−/−^ mice

**Figure 4:**
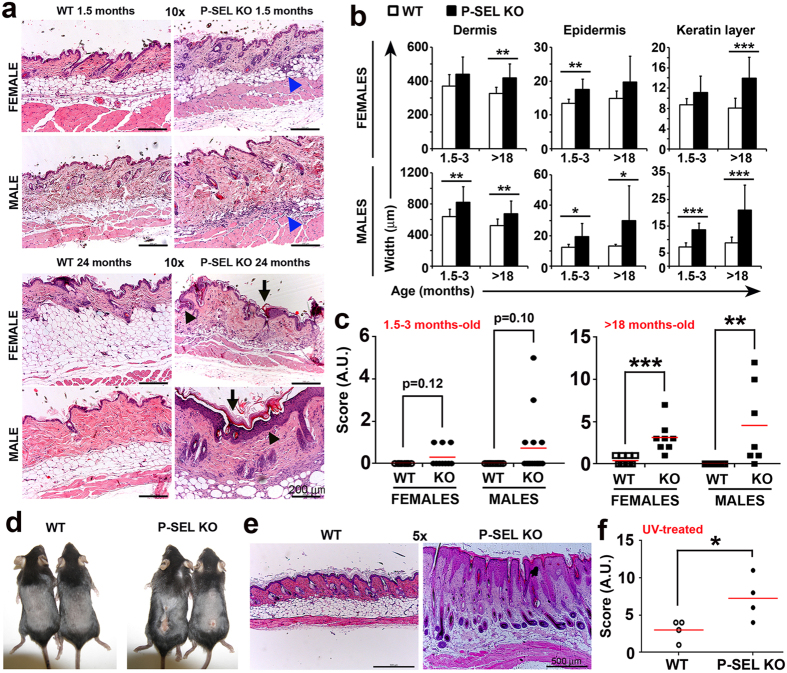
Histological alterations in the skin of *P-Sel*^−/−^ mice. (**a)** Photomicrographs (10×) of hematoxylin and eosin (H&E)-stained skin sections of 1.5 month-old (upper panels) and 24-month-old female and male WT and *P-Sel*^−/−^ mice (lower panels). Blue arrowheads show panniculitis. Black arrowheads show acanthosis; arrows show hyperkeratosis and keratin plugs. Scale bars represent 200 μm. (**b)** Quantification of dermis, epidermis and corneal layer width of WT and *P-Sel*^−/−^ mice. (**c)** Pathological activity index of skin samples obtained from WT and *P-Sel*^−/−^ mice. (**d)** Lesions developed in the back of UV-irradiated 3-month-old female WT and *P-Sel*^−/−^ mice. (**e)** Photomicrographs (5×) of representative skin sections of UV-irradiated 3-month-old female WT and *P-Sel*^−/−^ mice. n = 4 mice per genotype. Representative experiment of three independent replicates. Scale bars represent 500 μm. (**f)** Pathological activity index of skin samples obtained from UV-irradiated 3-month-old female WT and *P-Sel*^−/−^ mice. (**b,c,f**) Bars show the mean ± SD *p < 0.05; **p < 0.01; ***p < 0.005 by Student’s two tailed t test. n = 8–10 mice per genotype.

Histological examination of the skin revealed that, compared with WT counterparts, male and female *P-Sel*^−/−^ mice had a reduced hypodermal layer (lipoatrophy), frequently infiltrated by leukocytes (panniculitis) (Fig. 4a). We also found that, apart from a remarkable infiltration, >18-month-old *P-Sel*^−/−^ mice presented hyperproliferation of the epidermal layer (acanthosis), accumulation of keratin in the corneal layer (hyperkeratosis) and keratin plugs inside hair follicles (Fig. 4a, lower panels), described as murine lupus-like lesions^32^,^33^,^34^,^35^. Quantification of these observations by measuring the total thickness of the dermal, epidermal and corneal layers of WT and *P-Sel*^−/−^ mice showed that male and female *P-Sel*^−/−^ mice presented an enlarged dermis and incremented width of the epidermal and corneal layers in *P-Sel*^−/−^ mice that was more evident when mice were >18 months-old (Fig. 4b). Accordingly, the severity of the skin lesions estimated by grading scale was higher in young *P-Sel*^−/−^ mice (female *P-Sel*^−/−^ 0.30 ± 0.48 vs female WT 0.00 ± 0.00; male *P-Sel*^−/−^ 0.73 ± 1.44 vs male WT 0.00 ± 0.00) and remarkably more severe in the aged mice (>18 months-old: female *P-Sel*^−/−^ 3.13 ± 1.81 vs female WT 0.40 ± 0.52; male *P-Sel*^−/−^ 4.57 ± 4.83 vs male WT 0.00 ± 0.00) (Fig. 4c).

The exposure of 3–4-month-old female WT and *P-Sel*^−/−^ to UV radiation provoked an extensive dermatitis in *P-Sel*^−/−^ mice with ulcers in the exposed skin, but not in WT counterparts (Fig. 4d). Histopathological analysis revealed a severe epidermal lesion in female *P-Sel*^−/−^ mice consisting of acanthosis and hyperkeratosis, immune infiltration in the dermis, and deposition of extracellular matrix components in the hypodermal layer (Fig. 4e). Consequently, the severity of the lesions was significantly higher in UV-irradiated *P-Sel*^−/−^ mice (*P-Sel*^−/−^ 7.25 ± 2.99 vs WT 3.00 ± 1.41) (Fig. 4f).

### Kidney alterations and deposits of immune complexes in skin and kidneys of *P-Sel* ^−/−^ mice

**Figure 5:**
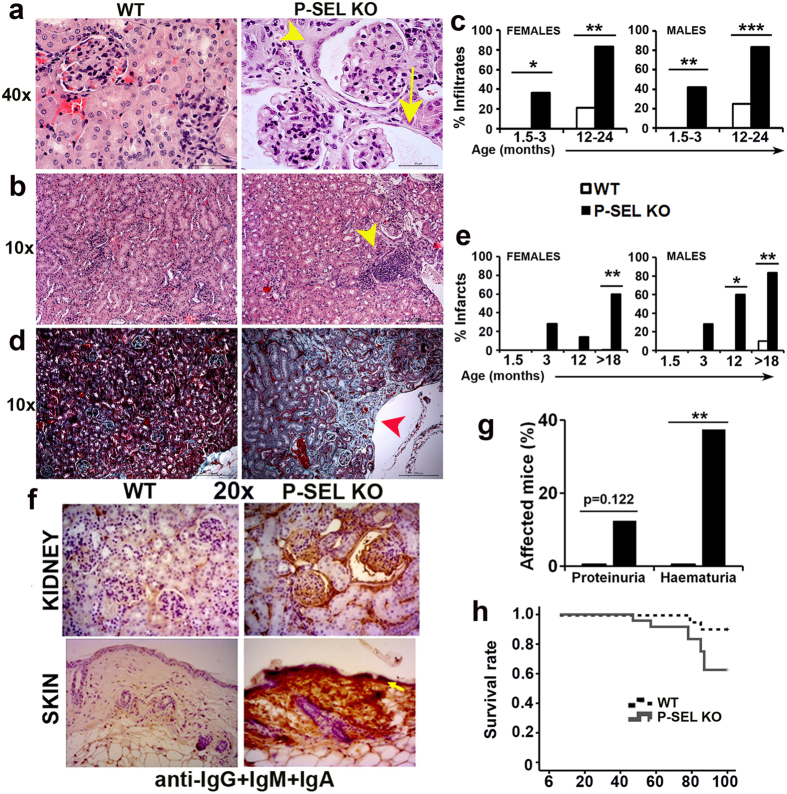
Kidney alterations and deposits of immune complexes in skin and kidneys of *P-Sel*^−/−^ mice. (**a)** Representative photomicrographs (40×) of H&E-stained kidney glomeruli of WT and *P-Sel*^−/−^ mice. Yellow arrow denotes a dilated Bowman’s space. Yellow arrowhead denotes a tubularized glomerulus. (**b)** Photomicrographs (10×) of kidney sections showing an immune infiltrate (yellow arrowhead) in *P-Sel*^−/−^ mice. (**c)** Prevalence of immune infiltration in WT and *P-Sel*^−/−^ mice (n = 8–10 mice per group). (**d)** Masson’s trichrome-stained kidney sections of WT and *P-Sel*^−/−^ mice (10×), showing healthy and infarcted tissue (red arrowhead), respectively. (**e)** Prevalence of renal infarcts in WT and *P-Sel*^−/−^ mice (n = 8–10 mice per group). (**f)** Representative photomicrographs of anti-IgM+ IgA+ IgG-stained kidney (upper panels) and skin (lower panels) sections (20×). n = 4–5 mice per group. Yellow arrow points to the dermoepidermal junction. (**g)** Frequency of proteinuria and hematuria in >12-month-old WT and *P-Sel*^−/−^ mice (n = 14–16 mice per group). (**h)** Kaplan-Meier survival curves for WT and *P-Sel*^−/−^ mice (n = 20 WT and 23 *P-Sel*^−/−^ mice); p = 0.033 by Mantel-Cox test. (**c,e,g**) *p < 0.05; **p < 0.01; ***p < 0.005 by Chi-square test.

Histological examination of kidney sections of WT and *P-Sel*^−/−^ mice showed that *P-Sel*^−/−^ mice had a high proportion of glomeruli with a dilated Bowman’s space (Fig. 5a). Additionally, some glomeruli presented tubularization of the Bowman’s capsule (Fig. 5a). We found also interstitial infiltrates in *P-Sel*^−/−^ mice (Fig. 5b), whose prevalence increased from 40% at 1.5–3 months of age to 84% in mice older than one year (12–24 months-old) (Fig. 5c), while only 20% of aged WT and none of the young WT mice presented interstitial infiltration.

We also observed that 30% of the 3-month-old *P-Sel*^−/−^ mice had infarcted foci (Fig. 5d). The prevalence of infarcts increased as mice grew older (Fig. 5e), reaching to 60% of females and 80% of males in the >18-month-old population of *P-Sel*^−/−^ mice.

We found deposits of immune complexes in the glomerular basal membrane in 100% of female and 25% of male *P-Sel*^−/−^ mice older than 18 months, but only in 25% of WT females and none in WT males (Fig. 5f). In the skin, we found deposits of immune complexes in 60% of male and 40% of female *P-Sel*^−/−^ mice over 18 months of age but in none of WT mice (Fig. 5f).

According to the structural renal damage and the immune complex deposition, we found that 13% of >18 months *P-Sel*^−/−^ mice developed proteinuria, while 38% of *P-Sel*^−/−^ mice develop hematuria (Fig. 5g).

### Reduced lifespan of *P-Sel* ^−/−^ mice

To analyze the impact of the autoimmune syndrome progression during the lifespan of *P-Sel*^−/−^ mice, we carried out a survival study with 20 WT and 23 *P-Sel*^−/−^ mice from 6 to 100 weeks of age. *P-Sel*^−/−^ mice started to die at week 47 and we observed a peak of mortality at around week 80. At week 100, 90% of WT mice remained alive whereas only 61% of the *P-Sel*^−/−^ mice were still alive (Fig. 5h).

### Decreased expression of P-Selectin in SLE skin biopsies

**Figure 6:**
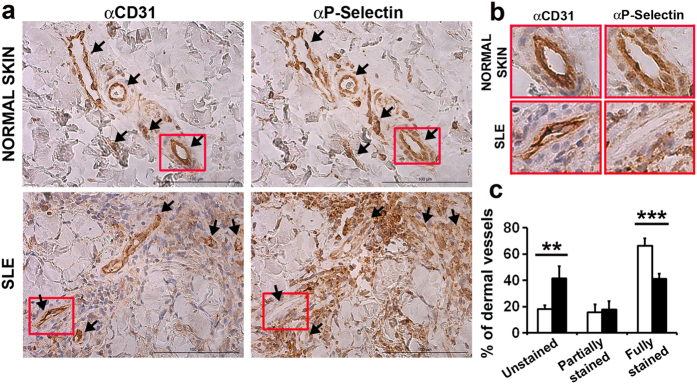
Decreased expression of P-Selectin in human SLE biopsies. (**a)** Representative photomicrographs (20×) of anti-CD31 and anti-P-Selectin stained skin biopsies of healthy donors and SLE patients (upper panels). Black arrows point blood vessels. (**b)** 200% magnification of representative blood vessels from the original images are represented (lower panels). (**c)** Classification and quantification of CD31+ dermal blood vessels according to the expression level of P-Selectin (healthy controls, n = 4; SLE patients, n = 4); bars show the mean ± SD. **p < 0.01 by Student’s two tailed t test.

To assess the relevance of P-Selectin in human lupus, we compared by immunohistochemical staining the expression of P-Selectin in endothelial cells of the dermal vessels of skin biopsies obtained from cutaneous lupus erythematosus (cLE) patients and healthy controls. We identified all the blood vessels (CD31^+^) in the whole biopsy and classified them into three categories depending on the expression of P-Selectin (Fig. 6a and b): (1) unstained, (2) partially stained, and 3) fully stained. We found a deep reduction of fully stained blood vessels in cLE biopsies, which were accompanied by a remarkable elevation in the percentage of negative vessels for P-Selectin expression (Fig. 6c). We also found unspecific binding to infiltrating leukocytes, that has been already reported by other authors^36^,^37^.

## Discussion

In this work, we show that the absence of P-Selectin breaks the immune tolerance and triggers the development of a progressive autoimmune lupus-like syndrome displaying most of the features previously described in lupus-prone mice. Importantly, we show that human biopsies of cutaneous lupus have reduced number of P-Selectin stained vessels.

Given that PSGL-1 deficient mice develop a scleroderma-like syndrome, we have analyzed whether the absence of P-Selectin, main ligand of PSGL-1, also triggered autoimmunity. We found the production of anti-dsDNA and anti-Sm autoandibodies, hallmarks of SLE in humans^38^. Interestingly, anti-Sm and anti-dsDNA autoantibodies are detected at an early age in *P-Sel*^−/−^ mice. The high rate of autoantibodies probably favours, as in human SLE, the formation and deposition of immune complexes in anatomic sites characterized by high blood pressure including capillaries of the skin and glomeruli, among others. Accordingly, we found a high rate of immune complexes deposition in renal glomeruli and skin of aged *P-Sel*^−/−^ which is a characteristic of both human and murine lupus^29^,^32^,^39^,^40^.

To understand the autoantibody production, we studied the impact of P-Selectin absence in the homeostasis of the immune system and analyzed the effector and regulatory leukocyte subsets in blood, spleen and skin of WT and KO mice, and found that the immune homeostasis is altered in *P-Sel*^−/−^ mice. We found higher percentage of germinal center B cell and Tfh subpopulations, indicating more reactive germinal centers in the spleen of young animals as well as reduced circulating TCD4^+^ and monocytic populations, in association with an increment in the B cell compartment. Interestingly, at the young age we found a reduction in the circulating tolerogenic IL-10-producing cDC and monocyte subsets, as well as in the IL-10^+^ B cell compartment (Breg), whose reduction has been recently implicated in the development of human SLE^41^. Although young *P-Sel*^−/−^ mice did not have altered the presence of Th1, Th2 and Th17 populations, reduction in the IL-10-producing CD4^+^ T cells implies an imbalanced Teff/Treg ratio, as it has been described for both murine and human SLE^42^. This reduction in IL-10^+^ cells could be explained by the lack of tolerogenic signal supplied by P-Selectin interaction with PSGL-1, as previously described^20^. Remarkably, we found a shift through a Th17 response with aging in the blood of *P-Sel*^−/−^ mice coinciding with the worsening of the disease, in agreement with the described role of PSGL-1 expression on Treg in the attenuation of persistent T cell activation during the immune response^43^. In fact, IL-17 promotes the recruitment of T cells, monocytes and granulocytes to the inflammatory foci, activates B cells and contributes to the synthesis of IgG and anti-dsDNA autoantibodies^44^. Accordingly, we report that, in *P-Sel*^−/−^ mice, the effector subpopulations of splenic CD4^+^ and CD8^+^ T cells were increased, with the consequent and relevant reduction among the naïve subset. SLE patients show reduced numbers of CD45RA^+^ naïve T cells and increased numbers of CD45RO^+^ memory T cells^45^. Interestingly, it has been described that mouse CD8^+^ CD122^+^ Treg have a central memory phenotype (CD44^high^CD62L^high^)^46^ what could explain the reduction of this compartment in the *P-Sel*^−/−^ mice, as well as the reduced populations of IL-10 producing T cells. In addition, this subset has been described to regulate T cell homeostasis and to suppress both autoimmune and alloimmune responses^46^.

Given that the skin is one of the main organs affected in lupus patients, we have also analyzed whether the dermal immune system is also altered in *P-Sel*^−/−^ mice. Our results indicate that dermal pDC subpopulation is increased from youth and that aging also increases the T cell subset. Importantly, coinciding with the appearance of the lupus skin lesions, we have found a high reduction in the dermal IL-10 producing immune cells and the FOXP3^+^ Treg population, together with an important expansion of the IL-17 producing T lymphocytes. Our data are in accordance with the expanded Th17 population and reduced Treg subset described for human patients^47^. It has been described that different mechanisms can be implicated in the breakage of the immunological tolerance^48^ and our work indicates that a defect in P-Selectin expression and function, probably through PSGL-1 interaction, could be implicated in maintaining the immune system immunity/tolerance balance. Dysfunction or low expression of P-Selectin and PSGL-1 could favor the use of alternative molecules for leukocyte extravasation and consequently, the loss of the tolerogenic signal triggered by PSGL-1/P-Selectin interaction.

The skin histological features described in this work for aged *P-Sel*^−/−^ mice, such as hyperkeratosis, acanthosis and immune cell infiltration, have been previously reported for MRL/lpr mice^33^,^49^. According with our data, it has been reported that P-Selectin or PSGL-1 deficiency in MRL/lpr mice results in an increase in the severity of dermatitis and glomerulonephritis^29^. We observed that UV irradiation could accelerate and intensify the appearance of the above mentioned lesions in 3–4-month-old *P-Sel*^−/−^ mice, whereas non lupus-prone strains are considered to be resistant to UV-induced DNA damage^38^. Interestingly, more than 50% of SLE patients show photosensitivity^38^,^50^,^51^.

Glomerulonephritis and renal involvement are the most common manifestations in SLE patients (40–70%)^52^. Leukocyte infiltration is a common feature shared by humans and lupus-prone mice^11^. Accordingly, we found leukocytic infiltration in both glomerular and tubulointerstitial compartments of the kidney in *P-Sel*^−/−^ mice. Additionally, *P-Sel*^−/−^ mice presented a high frequency of renal infarcts, indicating vascular dysfunction. Renal and systemic vasculitis have been described in patients and experimental models of SLE^27^ and, together with immune infiltration, have been associated with loss of renal function^53^. In agreement with our data, mice deficient for P-Selectin are more sensitive to glomerulonephritis^29^,^54^. Importantly, as a consequence of the renal damage, we found increased prevalence of proteinuria and hematuria in *P-Sel*^−/−^ mice.

We also report that the lifespan of *P-Sel*^−/−^ mice is reduced compared to WT animals, showing an augmented death rate at approximately 1 year of age (47 weeks), which is sharply increased at 80 weeks coinciding with disease worsening. This increased death along *P-Sel*^−/−^ mice could underestimate the prevalence of some clinical observations such as anti-DNA autoantibodies, skin lesions or proteinuria.

Finally, it has been reported that the lack of either P-Selectin or PSGL-1 in murine lupus models enhances both skin and renal inflammation^29^ and that the tissue expression of P- and E-Selectin in the MLR/lpr mice was not upregulated regarding the non-inflammed MRL^+/+^ mice^55^. Importantly, when analyzed in patients, although E-selectin expression was increased in skin biopsies^56^, according with our data in *P-Sel*^−/−^ mice, we found a reduction in the expression of P-Selectin in the dermal blood vessels of patients with cutaneous lupus. This important result does not concur with the higher levels of P-Selectin that have been traditionally associated with other autoimmune and inflammatory diseases like glomerulonephritis, rheumatoid arthritis, psoriasis or atopic dermatitis^37^,^57^,^58^. It has been reported that TNFα and LPS downregulated human P-Selectin^7^. The increased levels of TNFα in the serum of SLE patients are consistent with the low expression of P-Selectin that we found in the skin of lupus patients.

In summary, we show that P-Selectin expression is crucial for the immune system homeostasis and that its absence promotes the spontaneous development of a lupus-like syndrome in mice. Accordingly, patients with cutaneous lupus showed lower expression of P-Selectin in the endothelium of the dermal vessels, suggesting that the reduced expression of P-Selectin could be implicated in the pathogenesis of this disease. As SLE pathogenesis is not well understood and there is not a universally curative treatment for lupus in humans, it is very important to discover new molecules implicated in the development of the different forms of this disease that could be used as targets for new treatments. Our work will contribute to the understanding of lupus pathogenesis, and our future research will explore the role of P-Selectin in the development of human lupus and other autoimmune diseases. In addition, our work suggests that P-Selectin KO mice could be used as a new experimental model for *in vivo* assays to evaluate new treatments or combination of treatments against the progression of the disease that could prevent organ damage associated with SLE.

## Methods

### Mice

C57Bl/6 (WT) mice (The Jackson Laboratory) and C57Bl/6-*P-Sel*^−/−^ mice, kindly provided by Dr. D. Vestweber (Max Planck Institute for Molecular Biomedicine, Münster, Germany), were maintained at the Conventional Animal Facility of the School of Medicine of the Universidad Autónoma de Madrid (UAM) (register number ES-28079-0000097). Mice were sacrificed by cervical dislocation, and blood and internal organs were extracted for analysis. All experiments and breeding were performed in accordance with national and institutional guidelines for animal care (EU Directive 2010/63/EU for animal experiments). The experimental procedures were approved by the Director General de Medio Ambiente of Madrid (Ref: PROEX 69/14 and PROEX 162/15).

### UV radiation protocol

Three month-old WT and *P-Sel*^−/−^ mice were irradiated with an UVB lamp containing a set of six tubes (Phillips TL UV, 20 W; Royal Philips Electronics, Amsterdam, Netherlands) (ranging from 290 nm to 320 nm), being the energy output, at a distance of 15 cm, of 2.5 mW/cm^2^. Mice received three doses of 0.306 J/cm^2^ every other day for a week and an additional dose of 0.12 J/cm^2^ three days later. Skin was processed 24 hours after the last dose.

### Autoantibody assays

The presence of anti-nuclear antibodies (ANAs), the Crithidia assay for anti-dsDNA and the characterization and quantification of circulating autoantibodies were performed as previously described^22^. The cut-off point for positivity on ELISA for a particular autoantibody was determined as the mean value (X) plus two standard deviations (SD) obtained from sera of at least 100 WT mice with an age ranging from1.5 to 24 months. At least 8 mice per group of age were analyzed.

### Histopathological assessment and immunohistochemical (IHC) staining of mouse skin and kidney

Masson’s trichrome staining was performed with the Artisan Gomori’s Green Trichrome Stain kit (Dako; Glostrup, Denmark). Images were obtained with a Leica DM2500 light microscope and a Leica DFC450 camera. All sections were examined by a pathologist blinded to the sample origin.

Para-midline, upper back skin samples were evaluated blindly to obtain a semiquantitavive measurement by assigning a 3-criteria-based score^59^: acanthosis (0, normal; 1, slight thickening of epidermis; 2 and 3, presence of two or three layers of stratum spinosum cells, respectively; 4, presence of four or more layers of stratum spinosum cells); hyperkeratosis (0, normal; 1, one layer of keratin; 2, two layer thick of keratin; 3, minimum three-layer thick of keratin; 4, minimum three-layer thick of keratin and formation of a keratin’s plug); hypergrannulosis (0, normal; 1, moderate accumulation of granular dark material in the stratum granulosum; 2 and 3, two or more than three layers of granular dark material; 4, three or more layers of accumulated granular dark material overall section of skin). Finally, the total pathology score was calculated by adding the resulting values for the three criteria.

The presence of kidney ischemic events (infarcted areas) was assessed in Masson’s trichrome-stained sections.

Skin and glomerular immunoglobulin deposition were evaluated by IHC with an antibody cocktail against Fab regions of IgA, IgG, IgM (1/250) (Abcam; Cambridge, UK), following manufacturer’s instructions of the Universal LSAB+ Kit Rabbit/Mouse/Goat (DAB+) (Dako). At least 5 males and 5 females were evaluated per genotype and age.

### Urine analyses

Urinary protein and haemoglobin were determined using Combur Test M dipsticks (Roche; Basel, Switzerland). The cut-off for positivity in the proteinuria test was the lowest value in *P-Sel*^−/−^ mice not reached by any of the WT mice analyzed.

### Human skin samples

Skin biopsies from 4 chronic/subacute cutaneous lupus erythematosus patients and from 4 aged-matched healthy controls were obtained from the Pathology Department, Hospital de la Princesa (Madrid, Spain). The investigations were conducted in accordance with the principles of the Declaration of Helsinki and were approved by the Clinical Investigation Ethical Committee of the Hospital de la Princesa, Madrid, Spain (Register number: PI-654, date of approval 07-02-2013). Informed consent was obtained from all the patients and healthy controls.

### Immunohistochemistry of human skin sections

Consecutive tissue sections of sun-exposed skin biopsies underwent immunohistochemistry with the Dako REAL EnVision Detection System Peroxidase/DAB+kit (Dako), using as primary antibodies anti-human P-Selectin (10 μg/ml) (R&D Systems; Minneapolis, MN, USA) and anti-human CD31 (1/50) (Abcam). The whole biopsies were scouted for blood vessels in a blinded manner. Vessels were identified by positive staining by CD31, localized in the consecutive P-Selectin-stained section, and classified depending on the expression of P-Selectin as follows: 1) unstained, negative vessels; 2) partially stained; 3) fully stained blood vessels.

### Flow cytometry

Blood, skin and spleens from 1.5–3 month and >18 month-old male WT and *P-Sel*^−/−^ were analyzed. Spleens were dissected and mechanically disrupted in PBS 1X, 0.5% BSA, 5 mM EDTA. Skin samples were minced into ~1 mm^2^ pieces and digested with RPMI1640 medium complemented with 1 mg/ml collagenase A (Sigma-Aldrich; San Luis, MI, USA), 2.5 mg/ml dispase II (Roche; Basel, Switzerland) and 40 μg/ml DNase (Sigma). Cells were blocked with 1:200 Fc Block (BD Pharmingen; San Jose, CA, USA), labelled with the mix of antibodies recognising surface cell markers for 10 minutes at 4 °C and analyzed with a FACSCanto II (BD Pharmingen). For intracellular cytokine staining, after surface labelling, cells were fixed/permeabilized by 15 minute incubation with 2 ml of FACS Lysing Solution (BD Pharmingen), washed, incubated during 30 minutes at 4 °C with the cocktail of antibodies directed against intracellular cytokines and analyzed with a FACSCanto II.

Reagents: GL-7-eFLUOR660, CD3e-PE-Cy7 and CD11c-PE-Cy7 (eBioscience, San Diego, CA, USA); FAS-L-Biotin, CD3-APC, CD25-APC, CD45R/B220-APC, IFNg-APC, IL-4-APC, IL-17A-APC-Cy7, CD45.2-BV421, CD62L-PE and CD11b-FITC (BD Pharmingen); CD4-FITC, CD8a-PerCP, CD44-APC, CD8a-FITC, B220-APC-Vio770, MHC II-APC, and MHC II-PE (Miltenyi Biotec, Cologne, Germany); CD4-PE (Immunotools, Friesoythe; Germany); CXCR5-PE/Dazzle594, PD-1-BV421, IL-10-PerCP/Cy5.5, Gamma/delta TCR-PerCP/Cy5.5 and Streptavidin-PerCP (BioLegend, San Diego, CA, USA).

Gating strategy: in tissues, immune cells were identified as CD45^+^. CD11c^−^CD11b^+^ cells were gated as monocytes (peripheral blood) or macrophages (skin). CD11c^+^ cells were gated as DC: CD11c^+^ CD11b^+^ B220^−^ were gated as cDCs; and CD11c^+^ CD11b^−^B220^+^ were gated as pDCs. CD11c^−^B220^+^ cells, with low size/complexity index were considered B lymphocytes. T lymphocytes were gated as CD3^+^ cells. Follicular Th cells were gated as CD3^+^ CD4^+^ PD-1^high^CXCR5^high^. Germinal centre B cells were gated as CD11c^−^B220^+^ FAS-L^+^ GL-7^+^. Gamma/delta T cells were gated as CD3^+^γδ^+^.

### Statistical analysis

Statistical significance between two groups was calculated using two-tailed Student’s *t* test for parametric variables and Mann-Whitney’s U test for nonparametric variables. The chi-squared (df = 1) test was used for statistical comparison of frequencies. Mantel-Cox chi-squared (df = 1) test was used to analyze survival data. Differences were considered statistically significant with p < 0.05 (*) and highly significant at p < 0.01 (**) and p < 0.005 (***). All statistical analyses were performed using SPSS 15.0 program (IBM, Armonk, NY, USA). Skin pathology score graphic representation was performed with GraphPad Prism 6 (La Jolla, CA, USA).

## Additional Information

**How to cite this article**: González-Tajuelo, R. *et al*. P-Selectin preserves immune tolerance in mice and is reduced in human cutaneous lupus. *Sci. Rep.* **7**, 41841; doi: 10.1038/srep41841 (2017).

**Publisher's note:** Springer Nature remains neutral with regard to jurisdictional claims in published maps and institutional affiliations.

## References

[b1] Zarbock A., Ley K., McEver R. P. & Hidalgo A. Leukocyte ligands for endothelial selectins: specialized glycoconjugates that mediate rolling and signaling under flow. Blood 118, 6743–6751 (2011). 22021370 10.1182/blood-2011-07-343566 PMC3245201

[b2] McEver R. P. & Zhu C. Rolling cell adhesion. Annu Rev Cell Dev Biol 26, 363–396 (2010). 19575676 10.1146/annurev.cellbio.042308.113238 PMC3557855

[b3] Vestweber D. & Blanks J. Mechanisms that regulate the function of the selectins and their ligands. Physiol Rev 79, 181–213 (1999). 9922371 10.1152/physrev.1999.79.1.181

[b4] Wagner J. G. & Roth R. A. Neutrophil Migration Mechanisms, with an Emphasis on the Pulmonary Vasculature. Pharmacol Rev 52, 349–374 (2000). 10977867

[b5] Ley K. The role of selectins in inflammation and disease. Trends Mol Med 9, 263–268 (2003). 12829015 10.1016/s1471-4914(03)00071-6

[b6] Zhou C. . α1G T-type calcium channel selectively regulates P-selectin surface expression in pulmonary capillary endothelium. Am J Physiol Lung Cell Mol Physiol 299, L86–L97 (2010). 20435690 10.1152/ajplung.00331.2009 PMC2904100

[b7] Liu Z. . Differential regulation of human and murine P-selectin expression and function *in vivo*. J Exp Med 207, 2975–2987 (2010). 21149548 10.1084/jem.20101545 PMC3005233

[b8] Sanders W., Wilson R., Ballantyne C. & Beaudet A. Molecular cloning and analysis of *in vivo* expression of murine P-selectin. Blood 80, 795–800 (1992). 1379089

[b9] Weller A., Isenmann S. & Vestweber D. Cloning of the mouse endothelial selectins. Expression of both E- and P-selectin is inducible by tumor necrosis factor alpha. J Biol Chem 267, 15176–15183 (1992). 1378846

[b10] Hahne M., Jäger U., Isenmann S., Hallmann R. & Vestweber D. Five tumor necrosis factor-inducible cell adhesion mechanisms on the surface of mouse endothelioma cells mediate the binding of leukocytes. J Cell Biol 121, 655–664 (1993). 7683689 10.1083/jcb.121.3.655 PMC2119562

[b11] Bignon A. . CCR1 Inhibition Ameliorates the Progression of Lupus Nephritis in NZB/W Mice. J Immunol 192, 886–896 (2014). 24367031 10.4049/jimmunol.1300123

[b12] Lichtnekert J. . Activated protein C attenuates systemic lupus erythematosus and lupus nephritis in MRL-Fas(lpr) mice. J Immunol 187, 3413–3421 (2011). 21849682 10.4049/jimmunol.1101125

[b13] Laxminarayana D. Molecular insights into systemic lupus erythematosus pathogenesis. Clin Med Insights Pathol 7, 7–9 (2014). 24678246 10.4137/CPath.S14814 PMC3964202

[b14] Wu T. . Elevated urinary VCAM-1, P-selectin, soluble TNF receptor-1, and CXC chemokine ligand 16 in multiple murine lupus strains and human lupus nephritis. J Immunol 179, 7166–7175 (2007). 17982109 10.4049/jimmunol.179.10.7166

[b15] Morris D. . Variation in the upstream region of P-Selectin (SELP) is a risk factor for SLE. Genes Immun, 404–413 (2009). 19404301 10.1038/gene.2009.17 PMC2834331

[b16] Herrmann S. . The P-selectin gene is highly polymorphic: reduced frequency of the Pro715 allele carriers in patients with myocardial infarction. Human Molecular Genetics 7, 1277–1284 (1998). 9668170 10.1093/hmg/7.8.1277

[b17] Xia L. . P-selectin glycoprotein ligand-1-deficient mice have impaired leukocyte tethering to E-selectin under flow. J Clin Invest 109, 939–950 (2002). 11927621 10.1172/JCI14151 PMC150926

[b18] Rivera-Nieves J. . Critical role of endothelial P-selectin glycoprotein ligand 1 in chronic murine ileitis. J Exp Med 203, 907–917 (2006). 16567389 10.1084/jem.20052530 PMC2118267

[b19] McEver R. P., Moore K. & Cummings R. Leukocyte trafficking mediated by selectin-carbohydrate interactions. J Biol Chem 270, 11025–11028 (1995). 7538108 10.1074/jbc.270.19.11025

[b20] Urzainqui A. . Functional role of P-selectin glycoprotein ligand 1/P-selectin interaction in the generation of tolerogenic dendritic cells. J Immunol 179, 7457–7465 (2007). 18025190 10.4049/jimmunol.179.11.7457

[b21] Nuñez-Andrade N. . P-selectin glycoprotein ligand-1 modulates immune inflammatory responses in the enteric lamina propria. J Pathol 224, 212–221 (2011). 21432853 10.1002/path.2850

[b22] Pérez-Frías A. . Development of an autoimmune syndrome affecting the skin and internal organs in P-selectin glycoprotein ligand 1 leukocyte receptor-deficient mice. Arthritis Rheumatol 66, 3178–3189 (2014). 25132671 10.1002/art.38808

[b23] Singbartl K., Green S. & Ley K. Blocking P-selectin protects from ischemia/reperfusion-induced acute renal failure. FASEB J 14, 48–54 (2000). 10627279 10.1096/fasebj.14.1.48

[b24] Singbartl K. & Ley K. Protection from ischemia-reperfusion induced severe acute renal failure by blocking E-selectin. Crit Care Med 28, 2507–2514 (2000). 10921586 10.1097/00003246-200007000-00053

[b25] Rosenkranz A., Mendrick D., Cotran R. & Mayadas T. P-selectin deficiency exacerbates experimental glomerulonephritis: a protective role for endothelial P-selectin in inflammation. J Clin Invest 103, 649–659 (1999). 10074481 10.1172/JCI5183 PMC408121

[b26] Bullard D. . Acceleration and increased severity of collagen-induced arthritis in P-selectin mutant mice. J Immunol 163, 2844–2849 (1999). 10453030

[b27] Hickey M. J. Alterations in leucocyte trafficking in lupus-prone mice: an examination of the MRL/faslpr mouse. Immunol Cell Biol 81, 390–396 (2003). 12969327 10.1046/j.1440-1711.2003.01186.x

[b28] Kinoshita K. . Increases Survival in NZB/W F1 Mice. J Immunol 170, 5793–5798 (2003). 12759464 10.4049/jimmunol.170.11.5793

[b29] He X. . Deficiency of P-Selectin or P-Selectin Glycoprotein Ligand-1 Leads to Accelerated Development of Glomerulonephritis and Increased Expression of CC Chemokine Ligand 2 in Lupus-Prone Mice. J Immunol 177, 8748–8756 (2006). 17142777 10.4049/jimmunol.177.12.8748

[b30] Christensen S. . Toll-like receptor 7 and TLR9 dictate autoantibody specificity and have opposing inflammatory and regulatory roles in a murine model of lupus. Immunity 25, 417–428 (2006). 16973389 10.1016/j.immuni.2006.07.013

[b31] Rowland S. L. . Early, transient depletion of plasmacytoid dendritic cells ameliorates autoimmunity in a lupus model. J Exp Med 211, 1977–1991 (2014). 25180065 10.1084/jem.20132620 PMC4172228

[b32] Furukawa F. & Yoshimasu T. Animal models of spontaneous and drug-induced cutaneous lupus erythematosus. Autoimmun Rev 4, 345–350 (2005). 16081025 10.1016/j.autrev.2005.01.006

[b33] Furukawa F. . Dermatopathological studies on skin lesions of MRL mice. Arch Dermatol Res 276, 186–194 (1984). 6383229 10.1007/BF00414018

[b34] Guiducci C. . Autoimmune skin inflammation is dependent on plasmacytoid dendritic cell activation by nucleic acids via TLR7 and TLR9. J Exp Med 207, 2931–2942 (2010). 21115693 10.1084/jem.20101048 PMC3005224

[b35] Choi J.-Y. . Abrogation of Skin Disease in Lupus-Prone MRL/Faslpr Mice By Means of a Novel Tylophorine Analog. Arthritis Rheumatol 54, 3277–3283 (2006). 10.1002/art.22119 17009262

[b36] Dreßler J., Bachmann L., Koch R. & Müller E. Enhanced expression of selectins in human skin wounds. Int J Legal Med 112, 39–44 (1998). 9932741 10.1007/s004140050196

[b37] Miyazaki Y., Satoh T., Nishioka K. & Yokozeki H. STAT-6-Mediated Control of P-Selectin by Substance P and Interleukin-4 in Human Dermal Endothelial Cells. Am J Pathol 169 (2006). 10.2353/ajpath.2006.051211 PMC1698799 16877367

[b38] Furukawa F. Photosensitivity in cutaneous lupus erythematosus: lessons from mice and men. J Dermatol Sci 33, 81–89 (2003). 14581133 10.1016/j.jdermsci.2003.08.005

[b39] Savarese E. . Requirement of Toll-like Receptor 7 for Pristane-Induced Production of Autoantibodies and Development of Murine Lupus Nephritis. Arthritis Rheumatol 58, 1107–1115 (2008). 10.1002/art.23407 18383384

[b40] Rottman J. & Willis C. Mouse models of systemic lupus erythematosus reveal a complex pathogenesis. Vet Pathol 47, 664–676 (2010). 20448279 10.1177/0300985810370005

[b41] Menon M., Blair P. A., Isenberg D. A. & Mauri C. A Regulatory Feedback between Plasmacytoid Dendritic Cells and Regulatory B Cells Is Aberrant in Systemic Lupus Erythematosus. Immunity 44, 683–697 (2016). 26968426 10.1016/j.immuni.2016.02.012 PMC4803914

[b42] Shah K. . Dysregulated balance of Th17 and Th1 cells in systemic lupus erythematosus. Arthritis Res Ther 12 (2010). 10.1186/ar2964 PMC2888202 20334681

[b43] Angiari S. . Regulatory T cells suppress the late phase of the immune response in lymph nodes through P-selectin glycoprotein ligand-1. J Immunol 191, 5489–5500 (2013). 24174617 10.4049/jimmunol.1301235 PMC4627945

[b44] Ohl K. & Tenbrock K. Inflammatory Cytokines in Systemic Lupus Erythematosus. J Biomed Biotechnol 2011 (2011). 10.1155/2011/432595 PMC3196871 22028588

[b45] Fritsch R. D. . Abnormal Differentiation of Memory T Cells in Systemic Lupus Erythematosus. Arthritis Rheumatol 54, 2184–2197 (2006). 10.1002/art.21943 16802356

[b46] Liu J., Chen D., Nie G. D. & Dai Z. CD8+ CD122+ T-cells: a newly emerging regulator with central memory cell phenotypes. Front Immunol 6 (2015). 10.3389/fimmu.2015.00494 PMC4610204 26539191

[b47] Mak A. & Kow N. Y. The pathology of T cells in systemic lupus erythematosus. J Immunol Res (2014). 10.1155/2014/419029 PMC4017881 24864268

[b48] Hoffman R. T cells in the pathogenesis of systemic lupus erythematosus. Clin Immunol 113, 4–13 (2004). 15380523 10.1016/j.clim.2004.05.001

[b49] Deng G., Liu L., Bahjat F., Pine P. & Tsokos G. Suppression of skin and kidney disease by inhibition of spleen tyrosine kinase in lupus-prone mice. Arthritis Rheumatol 62, 2086–2092 (2010). 10.1002/art.27452 PMC2902591 20222110

[b50] Barbhaiya M. & Costenbader K. Ultraviolet radiation and systemic lupus erythematosus. Lupus 23, 588–595 (2014). 24763542 10.1177/0961203314530488

[b51] Ghoreishi M. & Dutz J. P. Murine models of cutaneous involvement in lupus erythematosus. Autoimmun Rev 8, 484–487 (2009). 19239927 10.1016/j.autrev.2009.02.028

[b52] Bertsias G., Cervera R. & Boumpas D. T. In *EULAR 2012* (ed. EULAR 2012) (Berlin, 2012).

[b53] Hamidou M. A., Audrain M. A., Masseau A., Agard C. & Moreau A. Anti-topoisomerase I antibodies in systemic lupus erythematosus as a marker of severe nephritis. Clin Rheumatol 25, 542–543, doi: 10.1007/s10067-005-0061-9 (2006). 16525896

[b54] Holdsworth S. & Tipping P. Leukocytes in glomerular injury. Semin Immunopathol 29, 355–374 (2007). 17938927 10.1007/s00281-007-0097-9

[b55] Harari O. A., Marshall D., McHale J. F., Ahmed S. & Haskard D. O. Limited endothelial E- and P-selectin expression in MRL/lpr lupus-prone mice. Rheumatology 40, 889–895 (2001). 11511758 10.1093/rheumatology/40.8.889

[b56] Belmont H., Buyon J., Giorno R. & Abramson S. Up-regulation of endothelial cell adhesion molecules characterizes disease activity in systemic lupus erythematosus. The Shwartzman phenomenon revisited. Arthritis & Rheumatism 37, 376–383 (1994). 7510492 10.1002/art.1780370311

[b57] Angiari S. Selectin-mediated leukocyte trafficking during the development of autoimmune disease. Autoimmun Rev 14, 984–995 (2015). 26117594 10.1016/j.autrev.2015.06.006

[b58] Segawa C. . *In situ* expression and soluble form of P-selectin in human glomerulonephritis. Kidney Int 52, 1054–1063 (1997). 9328945 10.1038/ki.1997.428

[b59] Bribes E., Galiegue S., Bourrie B. & Casellas P. Involvement of the peripheral benzodiazepine receptor in the development of cutaneous pathology in Mrl/Lpr mice. Immunol Lett 85, 13–18 (2003). 12505191 10.1016/s0165-2478(02)00177-3

